# Pharmacodynamic Analysis of Magnetic Resonance Imaging-Monitored Focused Ultrasound-Induced Blood-Brain Barrier Opening for Drug Delivery to Brain Tumors

**DOI:** 10.1155/2013/627496

**Published:** 2013-03-31

**Authors:** Po-Chun Chu, Wen-Yen Chai, Han-Yi Hsieh, Jiun-Jie Wang, Shiaw-Pyng Wey, Chiung-Yin Huang, Kuo-Chen Wei, Hao-Li Liu

**Affiliations:** ^1^Department of Electrical Engineering, Chang-Gung University, 259 Wen-Hwa 1st Road, Kwei-Shan, Tao-Yuan 333, Taiwan; ^2^Department of Diagnostic Radiology, Chang-Gung University and Memorial Hospital, 5 Fu-shin Street, Kwei-Shan, Tao-Yuan 333, Taiwan; ^3^Department of Medical Imaging and Radiological Sciences, Chang-Gung University, 259 Wen-Hwa 1st Road, Kwei-Shan, Tao-Yuan 333, Taiwan; ^4^Department of Neurosurgery, Chang-Gung Memorial Hospital, 5 Fu-shin Street, Kwei-Shan, Tao-Yuan 333, Taiwan

## Abstract

Microbubble-enhanced focused ultrasound (FUS) can enhance the delivery of therapeutic agents into the brain for brain tumor treatment. The purpose of this study was to investigate the influence of brain tumor conditions on the distribution and dynamics of small molecule leakage into targeted regions of the brain after FUS-BBB opening. A total of 34 animals were used, and the process was monitored by 7T-MRI. Evans blue (EB) dye as well as Gd-DTPA served as small molecule substitutes for evaluation of drug behavior. EB was quantified spectrophotometrically. Spin-spin (R_1_) relaxometry and area under curve (AUC) were measured by MRI to quantify Gd-DTPA. We found that FUS-BBB opening provided a more significant increase in permeability with small tumors. In contrast, accumulation was much higher in large tumors, independent of FUS. The AUC values of Gd-DTPA were well correlated with EB delivery, suggesting that Gd-DTPA was a good indicator of total small-molecule accumulation in the target region. The peripheral regions of large tumors exhibited similar dynamics of small-molecule leakage after FUS-BBB opening as small tumors, suggesting that FUS-BBB opening may have the most significant permeability-enhancing effect on tumor peripheral. This study provides useful information toward designing an optimized FUS-BBB opening strategy to deliver small-molecule therapeutic agents into brain tumors.

## 1. Introduction

Focused ultrasound beams (FUS) in the presence of circulating microbubbles can temporarily open the blood-brain barrier (BBB opening) of capillaries in the central nervous system (CNS) parenchyma [1–3]. Bursts of acoustic ultrasound induce microbubble cavitation in the vasculature, and the resultant shear stress temporarily disrupts tight junctions to enhance blood-brain permeability. This BBB-opening process can be carried out at moderate acoustic pressures to minimize adverse effects on vascularture and prevent damage to neurons [3–7], while facilitating localized delivery of chemotherapeutic agents from the vasculature to the pathological brain parenchyma and CNS [8–11]. Since more than 95% of the therapeutic agent normally cannot penetrate CNS tight junctions [12], this novel approach provides a unique opportunity for local delivery of therapeutic agents across the BBB and into the targeted site, thus opening a new frontier of CNS drug delivery. 

Brain tumors could potentially be treated by FUS-BBB opening to enhance chemotherapeutic agent delivery. In the United States, at least 18,000 patients are diagnosed with glioblastoma multiforme (GBM) each year, comprising more than half of the malignant primary brain tumors [13], and chemotherapy is an important treatment modality [14]. Recent preclinical studies showed that FUS-BBB opening can effectively enhance local deposition and concentration of chemotherapeutics including BCNU [11], liposomal doxorubicin [9, 15], and chemodrugs carried by novel nanocarriers [16].

Currently, FUS-mediated CNS drug delivery is monitored by magnetic resonance imaging (MRI) by intravenous (IV) injection of gadolinium diethylenetriamine penta-acetic acid (Gd-DTPA) contrast agent together with chemotherapeutic drug. The MRI signal intensity increase caused by the leakage of Gd-DTPA thus serves as an indicator to estimate drug concentration [4, 8, 9, 17]. Most studies have focused on analyzing the effects of FUS exposure parameters such as acoustic pressure amplitude, ultrasound frequency, pulse length, pulse repetition frequency, exposure duration, and microbubble dose on BBB opening [5, 18–23]. However, pharmacodynamic analysis including the dynamics and distribution of the specific molecular agent in the brain is critical for evaluating specific drug delivery. MRI could also be used for pharmacological endpoint evaluation using concurrently administered MR contrast agents as surrogate indicators of therapeutic drug concentrations. Although the kinetics of contrast agent permeability of a defective blood-brain barrier have been measured using MR compartment modeling [24–26], these studies were performed in normal animal brains, and so far the detailed pharmacodynamic behavior of contrast agents after FUS-BBB opening remains uncertain in brain tumors. 

The purpose of this study was to conduct an MRI pharmacodynamic analysis of FUS-BBB opening in brain tumors in an animal model. Injected Gd-DTPA contrast agent was used to characterize pharmacodynamic changes as a function of time after BBB opening, in both normal and brain-glioma animals. We also attempted to establish the correlation between deposition of Gd-DTPA by *in vivo* semiquantification and the quantitation of another surrogate, Evans blue dye, by spectrophotometry after sacrifice. Finally, we evaluated the pharmacodynamic changes affected by FUS-BBB opening in various grades of gliomas. 

## 2. Methods

### 2.1. FUS Setup

**Figure 1:**
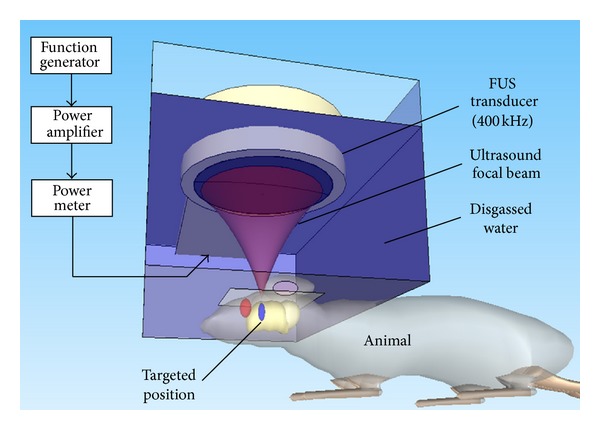
Schematic showing the experimental setup of the focused ultrasound exposure system.

A focused ultrasound transducer was used to generate ultrasound focal energy (IMASONIC, France; diameter = 60 mm, radius of curvature = 80 mm, frequency = 400 kHz, and electric-to-acoustic efficiency = 70%) (Figure 1). An arbitrary function generator (33120A, Agilent, Palo Alto, CA and DS345, Stanford Research Systems, Sunnyvale, CA) was used to generate the driving signal, which was then fed into a radiofrequency power amplifier (150A100B, Amplifier Research, Souderton, PA). The focal zone distribution of the intensity of the ultrasound field was measured in an acrylic water tank filled with deionized, degassed water. The measured diameter of the half-maximum pressure amplitude was 2 mm, and the length of the produced focal zone was 15 mm. Animals underwent isofluorane anesthesia before ultrasound treatment. The animal was laid prone and placed directly under an acrylic water tank (with a window of 4 × 4 cm^2^ at its bottom sealed with a thin film to allow entry of the ultrasound energy), using ultrasound gel to fill the interspaces between the animal head and the thin-film window. SonoVue SF6-coated ultrasound microbubbles (2–5 *μ*m mean diameter, 2.4 *μ*L/kg; Bracco Diagnostics Inc.) were IV administered by burst injection with 0.1 mL of saline solution containing 0.01 mL heparin. After injecting the microbubbles, burst-tone mode ultrasound at a pressure of 0.4 MPa (peak negative value; measured in the free-field) was delivered to the brain with the center of the focal zone positioned at a penetration depth of 4-5 mm under the scalp (burst length = 10 ms, pulse repetition frequency = 1 Hz, and total sonication duration = 90 s).

### 2.2. Animal Experiment Design

All animal experiments were approved by the Institutional Animal Care and Use Committee of Chang Gung University and adhered to the experimental animal care guidelines. A total of 34 animals (male Sprague-Dawley rats (250–300 g)) were used, including normal (*n* = 18) and tumor animals (*n* = 16). Experiments were divided into two groups. In group 1, the aim was to confirm the correlation between Gd-DTPA leakage (concentration measured by relaxometry) and Evans blue (EB) dye (concentration measured spectrophotometrically) after FUS-BBB treatment. Subgroups included (1) normal rats (*n* = 18) and (2) tumor rats (*n* = 4), and the first subgroup underwent FUS-BBB opening. Subgroups were confirmed by dynamic contrast-enhanced (DCE) MRI with Gd-DTPA (molecular weight = 938 Da). In addition, EB dye (molecular weight = 960 Da) was IV injected into the animals, and the amount of EB deposited in the brain was quantified spectrophotometrically (procedure described below). In the first subgroup of group 1, contrast-enhanced T_1_-weighted imaging was first performed to estimate Gd-DTPA concentration after BBB opening, followed by T_2_-weighted imaging to provide a reference of tumor morphology. The second subgroup underwent the same scanning process without FUS induced.

**Figure 2:**
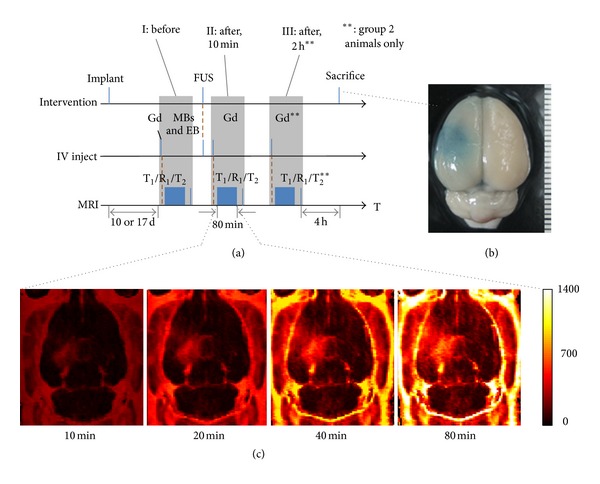
(a) Experimental protocol for 1st- and 2nd-group animal experiments. MRI images were acquired in time slots I/II for group 1 animals and in time slots I/II/III for group 2 animals; double asterisks (**) indicate group-2 experiments only. (b) FUS-exposed brain of a normal animal with Evans blue extravasations to identify the location of BBB opening. (c) R_1_ accumulating map showing Gd-DTPA accumulation in the BBB-opening location of a normal animal over time.

 In experimental group 2, our aim was to monitor the increase in Gd-DTPA accumulation in tumor-bearing animals after conducting FUS-BBB opening. Animals were divided into two subgroups: (1) animals receiving FUS exposure 10 days after tumor implantation (tumor volume typically <0.05 cm^3^) with FUS-BBB opening (*n* = 6) and (2) animals receiving FUS exposure 17 days after tumor implantation (tumor volume typically >0.05 cm^3^) with FUS-BBB opening (*n* = 6). Tumor volume was measured by T_2_-weighted MRI. In group 2, animals were subjected to three 80-minute-long MR relaxometry-based imaging sessions (before FUS exposure, and 10-min and 120-min after FUS exposure). Detailed experimental procedures are shown in Figure 2.

### 2.3. Rat Brain Glioma Model

C6 glioma cells were harvested by trypsinization and cultured at a concentration of 1 × 10^5^ cells/mL for implantation. For intracranial injection into the striatum of rat brains, cells were washed once with phosphate buffered saline (PBS). Male Sprague-Dawley rats (250–300 g) were anesthetized by intraperitoneal administration of ketamine (100 mg/kg) and immobilized on a stereotactic frame. A sagittal incision was made through the skin overlying the calvarium, and a small dental drill was used to make a hole in the exposed cranium, 0.5 mm anterior and 3 mm lateral to the bregma on the left side of skull. C6 cell suspension (5 mL) was injected at a depth of 4.5 mm from the brain surface. The injection was performed over a 10-minute period, and the needle was withdrawn over another 2 minutes. Ten days after implantation, tumor sizes were measured by MRI.

### 2.4. Spectrophotometric Quantitation of Evans Blue Dye

EB dye (3% in saline) was IV injected (2 mg/kg), and the animals were sacrificed two hours later. All animals were first deeply anesthetized with 10% chloral hydrate and infused with heparinized saline through the cardiac ventricle until colorless infusion fluid was obtained from the atrium. After the rats had been sacrificed by decapitation, the hemispheres of the brain were separated along the transverse suture. Then both hemispheres were weighed and placed in formamide (1 mL/100 mg) at 60°C for 24 h. The sample was centrifuged for 20 mins at 14,000 rpm. The concentration of dye extracted from each brain was determined spectrophotometrically at 620 nm and was compared with a standard graph created by recording optical densities from serial dilutions of EB in 0.9% sodium chloride solution. The EB tissue content was quantified using a linear regression standard curve derived from seven concentrations of the dye. 

### 2.5. MRI

For *in vitro* measurements, Gd-DTPA (Omniscan, 0.3 mL/kg, Magnevist) was diluted with physiological saline to 0.12, 0.24, 0.49, 0.97, 1.96, and 3.9 *μ*M. Circular wells (inner diameter = 5 mm) were filled with 200 *λ* of contrast agent sample or physiological saline as control and were placed in the MR scanner (Clinscan, Bruker, Germany; 7 Tesla). Spin-lattice relaxivity maps were calculated from two T_1_-weighted images with different flip angles (gradient recalled echo sequence, TR/TE = 2.3 ms/0.76 ms, slide thickness = 0.8 mm, matrix = 132 × 192, and flip angle = 5°/20°). The correlation between R_1_ (= 1/T_1_) mapping and Gd-DTPA concentration was determined [27]. 

In the animal experimental group, FUS-induced BBB opening was monitored by MRI with a 7-Tesla magnetic resonance scanner (Bruker ClinScan, Germany) and a 4-channel surface coil. The mouse was placed in an acrylic holder, positioned in the center of the magnet, and anesthetized with isoflurane gas (1-2%) at 50–70 breaths/min during the entire MRI procedure.

In the first experimental group, the distribution and dynamics of Gd-DTPA leakage were investigated immediately after conducting FUS-BBB opening. After FUS-BBB opening, animals were immediately relocated into the MR scanning room, and contrast-enhanced T_1_-weighted images with different flip angles were acquired to calculate spin-lattice relaxivity maps by transferring two images with different flip angles (gradient recalled echo sequence, TR/TE = 2.3 ms/0.76 ms, slice thickness = 0.8 mm, slice number = 14, matrix = 132 × 192, and flip angle = 5°/20°). Images were sequentially acquired over 80 min with a time interval of 60 seconds for area under the curve (AUC) calculation. Upon completion of the 10th acquisition, a diluted bolus of Gd-DTPA was IV injected through a catheter at an infusion rate of 6 mL/s. In the second experimental group, three sets of Gd-DTPA-leakage distribution/dynamics were investigated, including (I) before FUS exposure, (II) immediately after FUS exposure, and (III) two hours after FUS exposure. Immediately after conducting FUS-BBB opening, turbo spin echo (TSE) T_2_-weighted images were obtained as a reference to identify the tumor region (repetition time (TR)/echo time (TE) = 2540/41 ms, FOV = 34 × 40 mm^2^, in-plane resolution = 0.4 × 0.3 mm^2^, and slice thickness = 0.6 mm). 

### 2.6. MR Analysis of Gd-DTPA Accumulation and Distribution after FUS-BBB Opening

In R_1_-map analysis, a region of interest (ROI) was selected and compared with the non-enhanced contralateral brain to determine the increase in Gd-DTPA concentration caused by BBB opening. AUC maps were then transferred from a series of time-dependent R_1_ maps (up to 80 min) to determine pharmacodynamic characteristics of Gd-DTPA for comparison with the dynamics of EB dye permeability. Thus, the total area (AUC) is given by the following equation:

(1) $$\begin{array}{c} {\text{AUC}}_{80\;\min}=\frac{\int_{}^{}Cpt\cdot dt}{V}, \end{array}$$

where *Cpt* are vertical segments under the Gd-DTPA concentration curve area and *V* is total ROI volume.

In experimental group 2, ROIs were selected in the targeted tumor area which was based on the tumor dimensions defined in T_2_ images (the same ROI as in the contralateral brain was selected). The distribution and dynamics of Gd-DTPA leakage were evaluated for different tumor sizes including 10 days after implantation (typically <0.05 cm^3^) and 17 days after implantation (typically >0.05 cm^3^) and were divided by the tumor dimension. ROI including the entire tumor and the contralateral area were selected. Moreover, in order to evaluate the homogeneity of Gd-DTPA leakage, tumors with dimension >0.05 cm^3^ were further divided into tumor core (inner half of the area) and tumor peripheral (outer half of the area), based on T_2_ images.

### 2.7. Histology

Albumin-bound EB dye was IV injected as a bolus immediately after sonication. BBB opening was quantified as extravasation of EB. Tumor model animals were sacrificed about 2 h after sonication and MR scanning. Brain samples were serially sectioned (2 *μ*m thickness) using the same slice direction as in MRI analysis. Representative sections were stained with hematoxylin and eosin (HE). Tumor morphology was histologically evaluated. 

## 3. Results

BBB opening was clearly evidenced by staining with EB dye. A typical image of a normal BBB-opened brain stained with EB dye is shown in Figure 2(b). In addition, a series of R_1_ maps obtained at different time points after FUS-BBB opening demonstrated the dynamic change in Gd-DTPA accumulation in a normal brain, with particularly high leakage at the sonication site (Figure 2(c)).

**Figure 3:**
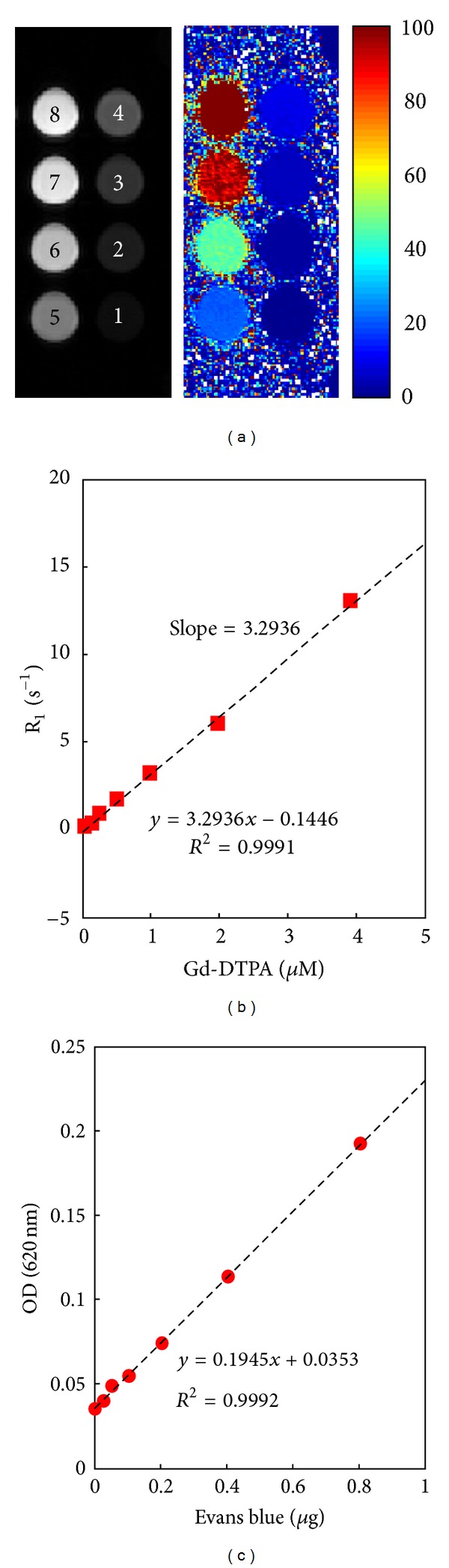
(a) T_1_ image and corresponding R_1_ map for the *in vitro* Gd-DTPA phantom at increasing concentrations (1 and 2: water; 3: 0.12 *μ*M; 4: 0.24 *μ*M; 5: 0.49 *μ*M; 6: 0.97 *μ*M; 7: 1.96 *μ*M; 8: 3.9 *μ*M). (b) Dependence of R_1_ on Gd-DTPA concentration; relaxivity was estimated as about 3.2936 s^−1 ^mM^−1^. (c) Calibration curve of spectrophotometrically determined EB concentration. EB concentrations ranging from 0 to 0.8 *μ*g were tested, resulting in O.D. readings of 0.035 to 0.19 at 620 nm.

R_1_ relaxivity of Gd-DTPA and ELISA measurements of EB dye concentration were calibrated *in vitro*. The detected R_1_-signal increased in a highly linear manner with Gd-DTPA concentration (input concentrations of 0, 0.25, 0.5, 1, 2, and 4 *μ*M) as shown by the calibration curve (*r* ^2^ = 0.9991) (Figure 3(b)). The relaxivity of Gd-DTPA contrast agent was found to be 3.3 at 7 Tesla. The detected ELISA signal also increased in a highly linear manner with EB concentration (Figure 3(c); *r* ^2^ = 0.9992). These calibration curves thus allowed precise quantitation of Gd-DTPA and EB deposition in the brain. 

**Figure 4:**
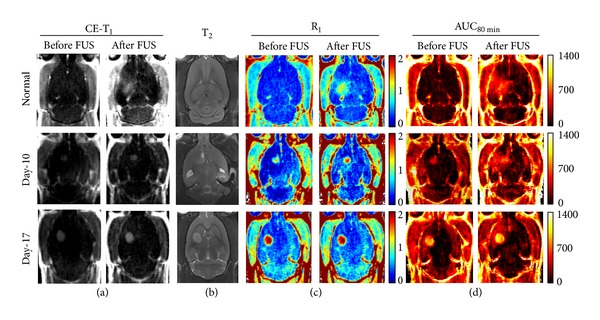
Typical MRI for normal animals (upper) as well as animals after 10-day (middle) and 17-day (bottom) tumor implantations. (a) Contrast-enhanced T_1_ images before and after FUS exposure. (b) T_2_ images (after FUS). (c) R_1_ maps before and after FUS exposure. (d) Area under the R_1_ curve over 80 minutes (denoted as AUC_80 min⁡_) before and after FUS exposure.

FUS-induced BBB opening was verified by CE-MRI. Typical CE-T_1_ images, T_2_ images, R_1_ maps, and AUC maps in normal, 10-day glioma, and 17-day glioma animals are shown in Figure 4. In normal animals, the BBB-opened area was clearly visible in T_1_-weighted images. T_2_-weighted images did not show any evidence of FUS-induced damage at the target location at pressure amplitudes of 0.4 MPa (Figure 4). In the first subgroup of experimental group 1, the R_1_-map signal of the BBB-opening area was increased from 0.1 to about 1.2 by FUS, and AUC maps showed an increase in accumulation of Gd-DTPA deposition from 20 to about 600.

In tumor-bearing animals, the FUS-induced BBB area clearly covered the tumor tissue (Figure 4; small (10-day) or large (17-day) tumors). Sonication resulted in increased Gd-DTPA accumulation in the tumor and in the peripheral BBB-opened area as evidenced by signal enhancement in the R_1_-map images. AUC maps showed maintained high staining intensities after Gd-DTPA injection. In the small (10-day) tumors, FUS resulted in an increase of R_1_ signal from 1 to about 1.5 s^−1^ and an increase in Gd-DTPA deposition of about 200 (from 400 to 600). However, in large (17-day) tumors, FUS did not lead to a significant change in the R_1_-signal, which increased from 1.9 to 2 s^−1^, or the AUC value which increased by only about 50 (from 1000 to 1050). 

**Figure 5:**
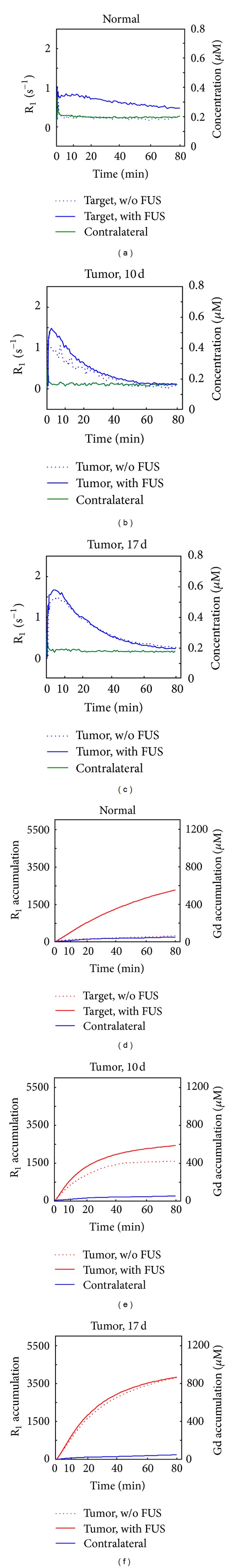
(a)–(c) R_1_ change as a function of time before and after FUS exposure for normal animals, 10-day and 17-day tumor animals. (d–f) The corresponding R_1_ accumulation as a function of time over 80 minutes for (a)–(c).

The kinetics of Gd-DTPA accumulation were evaluated after a single sonication treatment in thirty animals (normal rats: *n* = 18; small-tumor model: *n* = 6; large-tumor model: *n* = 6). An ROI from the BBB-opened area on T_2_-weighted images (target) and the corresponding ROI from the contralateral brain (contralateral) were used to infer Gd-DTPA concentration from the R_1_ signals and the AUC over time in the 10-day tumors (volume < 0.05 cm^3^), 17-day tumors (volume > 0.05 cm^3^), or normal controls after sonication (Figure 5). Figures 5(a)–5(c) showed the comparison of changes in R_1_ as a function of time (from 0 to 80 min) for three typical animals. When considering the peak value over the whole scanning process, FUS caused the highest enhancement in R_1_ signal of contrast agent in normal tissue (from 0.2 to 0.84; Figure 5(a)). Sonication also led to a large increase in R_1_ signal in 10-day tumor, from 0.86 to 1.48 s^−1^ (Figure 5(b)). However, the already high permeability of 17-day tumor to Gd-DTPA was not significantly increased by FUS, from 1.49 to 1.62 s^−1^ (Figure 5(c)).

The corresponding AUC (accumulation of R_1_) as a function of time (from 0 to 80 min) in these three animals is shown in Figures 5(d)–5(f). In the normal animal, total Gd-DTPA accumulation in the BBB-opening area was increased from 44.2 to 552.6 pmol by FUS (Figure 5(d)). Gd-DTPA accumulation in the 10-day tumor increased from 411.7 to 577.5 pmol, compared to only about 73 pmol over time in the contralateral control hemisphere (Figure 5(e)). However, Gd-DTPA accumulated to about the same high value with and without FUS (863.8 versus 867.3 pmol) in the 17-day tumor (Figure 5(f)).

**Figure 6:**
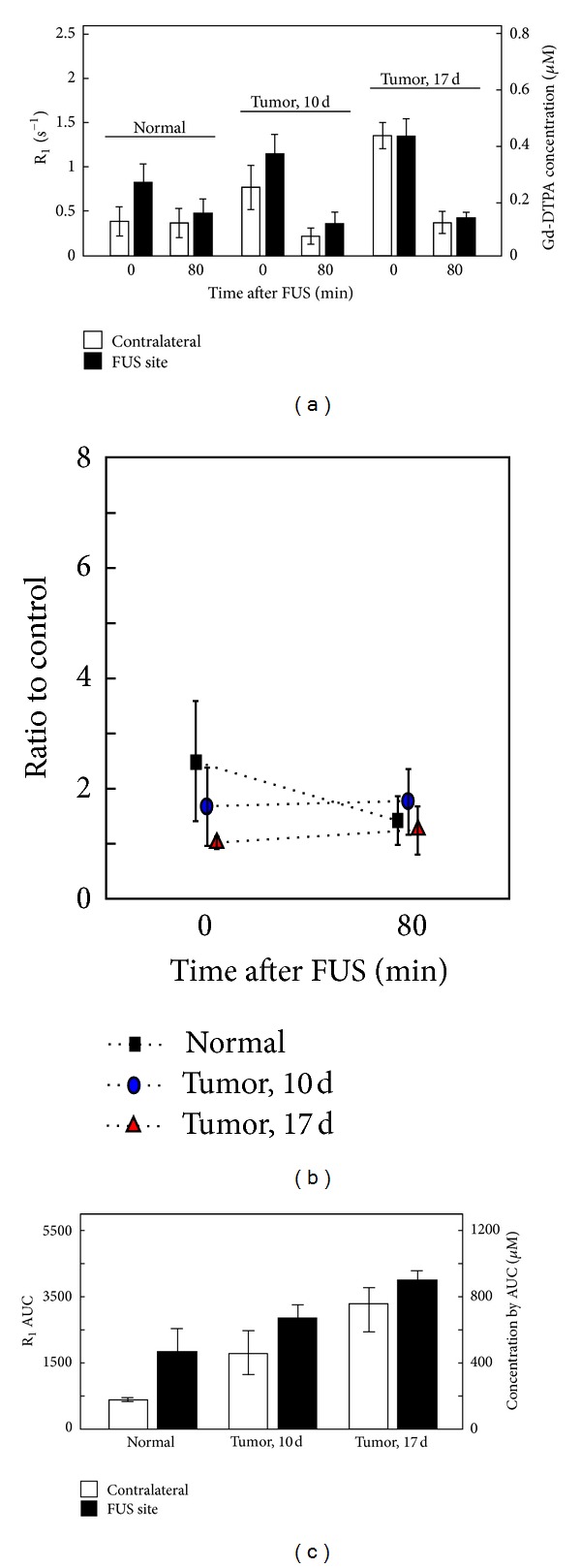
(a) Instantaneous R_1_ measured immediately or 80 minutes after FUS exposure in normal animals, 10-day and 17-day tumor animals. (b) Ratio of instantaneous R_1_ values, immediately and 80 min after FUS. (c) Corresponding R_1_ AUC of (a). Images were acquired in time slot II.

Gd-DTPA levels on the FUS-treated and contralateral side were also evaluated at either 10 min or 80 min after a single sonication treatment (Figure 6). The Gd-DTPA signal intensity increased in the BBB-opening area 10 min after sonication as evidenced by an increase in the R_1_ signal from 0.3863 to 0.8313 s^−1^ for control and from 0.77 to 1.15 s^−1^ for small-tumor animals. However, the contrast agent signals in the FUS-enhanced large tumors were the same as for the contralateral region (about 0.136 s^−1^). All R_1_ signals in these brain tissues returned to baseline (about 0.428 s^−1^) at 80 min after sonication. The ratio of R_1_ between the FUS-exposed and control areas went from 2.48 s^−1^ at 10 min to 1.4 s^−1^ at 80 min in normal tissue and from about 1.8 to 1 s^−1^ for the tumor-bearing animals (Figure 6(b)). The AUC at 10 min and 80 min was compared between control and BBB-opened brain regions. At 10 min after sonication, the accumulated Gd-DTPA concentration of the BBB-opened area increased to 465.99 *μ*M, compared to 667.34 *μ*M for the small tumor and a limited increase to 896 *μ*M for the large tumor (Figure 6(c)).

**Figure 7:**
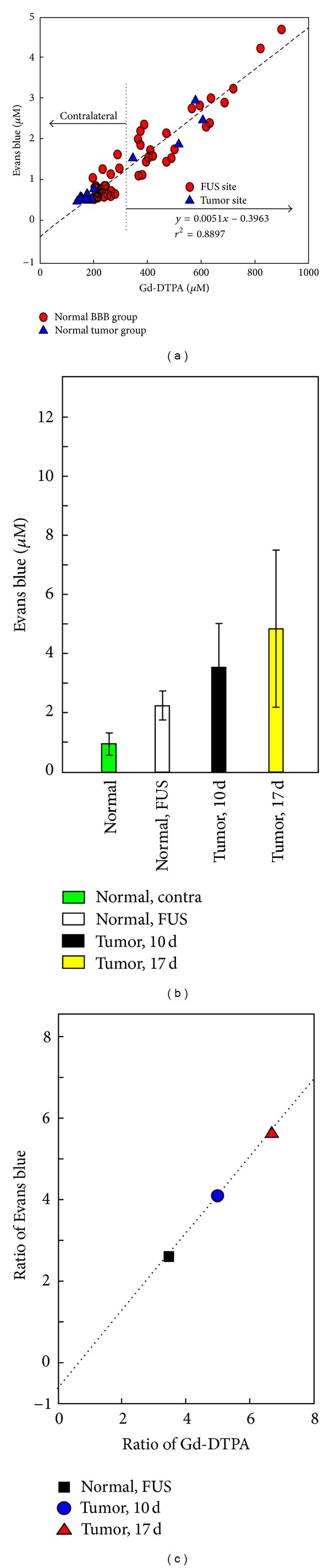
(a) Correlation between quantified Evans blue concentration and R_1_-estimated Gd-DTPA concentration in normal (red circle) and tumor (blue triangle) brains. Left arrow: contralateral side and right arrow: with FUS (red circle); normal tumor (blue triangle). (b) Evans blue after FUS exposure in the normal contralateral region, normal FUS targeting region, 10-day tumor region after FUS, and 17-day tumor region after FUS. (c) Correlation of the ratios of FUS: contralateral concentrations of Evans blue and Gd-DTPA in normal and tumor-bearing brains.

Next we evaluated the correlation between EB leakage (Figure 7(a)) and Gd-DTPA accumulation (estimated by the AUC) in the same region of the brain. We found that the accumulated distribution of Gd-DTPA (i.e., AUC) was highly correlated with the distribution of EB (*r* ^2^ = 0.8897) (Figure 7(b)). Thus, R_1_-based pharmacodynamic analysis provided a reasonable map of the permeability of the BBB-disrupted region to EB dye over time. EB dye and Gd-DTPA accumulation showed the same tendency of higher overall permeability in tissues of large tumors and less dependence on FUS treatment, as evidenced from the ratios of accumulation between contralateral and FUS-treated regions (Figure 7(c)).

**Figure 8:**
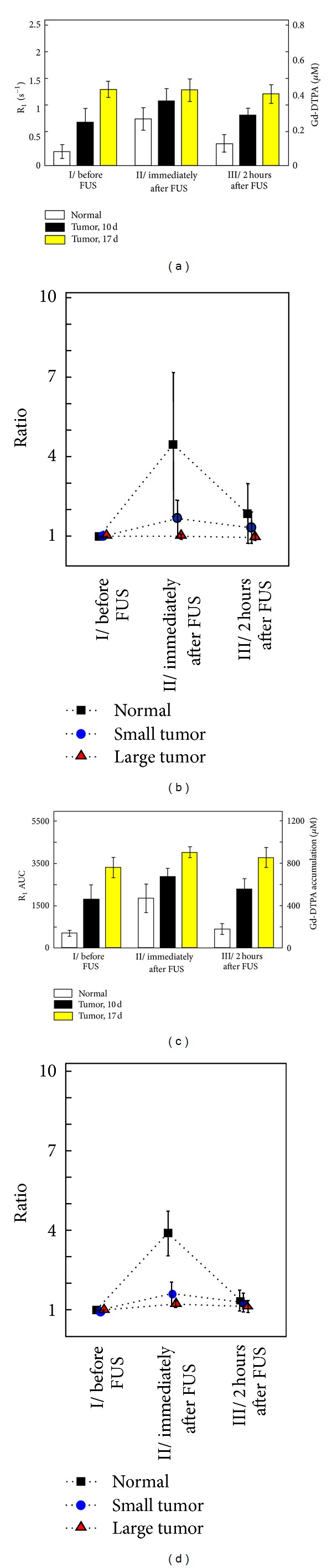
(a) Instantaneous R_1_ values of the normal animals, 10-day and 17-day tumor animals at MRI acquisition time slots I, II, and III. (b) Ratio of instantaneous R_1_ to the value in time slot I. (c) Corresponding R_1_ AUC of (a). (d) Ratio of R_1_ AUC to the value in time slot I.

Next, we analyzed Gd-DTPA deposition dynamics in experimental group 2 animals by AUC analysis for three individual time slots: (I) before FUS, (II) immediately after FUS, and (III) 2 hours after FUS. Gd-DTPA concentration and accumulation in the target area presented the same trend at all three time points (Figure 8). As before, we observed a transient peak of R_1_ and the AUC in the BBB-opened brain just after sonication (Figures 8(a) and 8(c)). However, the increase in the ratio of R_1_ or AUC again differed between the normal and tumor-bearing animals (Figures 8(b) and 8(d)). The ratio was highest in the normal BBB-opening area (R_1_ signal 4.4 times than before FUS; AUC: 3.7 times), followed by the small-tumor model (R_1_ signal: 1.7 times; AUC: 1.6 times), with no significant change in the large tumor, confirming our previous observations that FUS did not significantly further affect permeability in large tumors. These ratios subsequently decreased at the time point 2 hours after FUS induction, returning to approximately the same values of DCE-MRI as originally observed before sonication. This result implied that at 2 hours, the BBB-opening area had recovered to the same baseline permeability level to contrast agent as prior to sonication.

**Figure 9:**
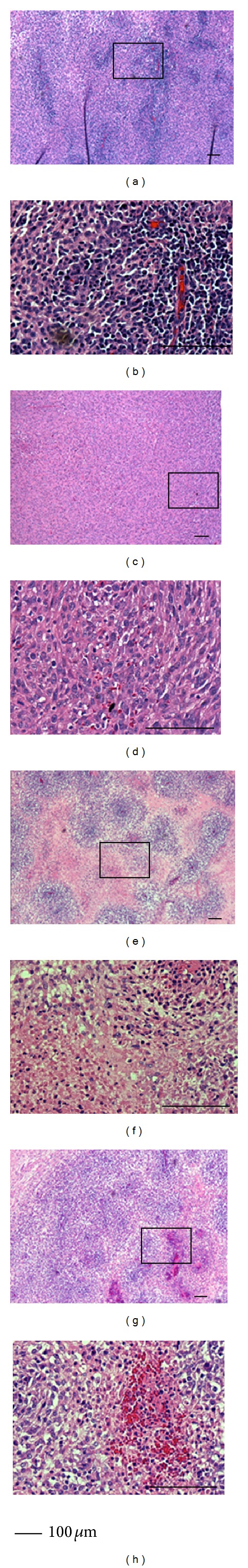
HE staining. (a) and (b) Small (10-day) tumor tissue without FUS exposure, 40x and 200x. (c) and (d) Small (10-day) tumor tissue with FUS exposure, 40x and 200x. (e) and (f) Large (17-day) tumor tissue without FUS exposure, 40x and 200x. (c) and (d) Large (17-day) tumor tissue with FUS exposure, 40x and 200x.

HE staining of tumors 10 days after implantation showed even staining without scattered red blood cells in the absence of FUS (Figures 9(a) and 9(b)). Tumor cells were characterized by dense nuclear distribution, and only tiny areas of gliosis infiltrated with chronic inflammatory cells and some hemorrhaging were found (Figures 9(c) and 9(d)). HE staining of tumors 17 days after implantation revealed a number of regions with extensive apoptosis and cavities in the core of the tumor, and hemorrhagic structures with scattered and spreading erythrocytes could be observed around discontinuous vasculature (Figures 9(e) and 9(f)), supporting our findings of high permeability of 17-day tumors based on observation of Gd-DTPA deposition. This structure did not change significantly after sonication (Figures 9(g) and 9(h)) with hemorrhagic regions remaining similar to those in the unexposed tumor.

**Figure 10:**
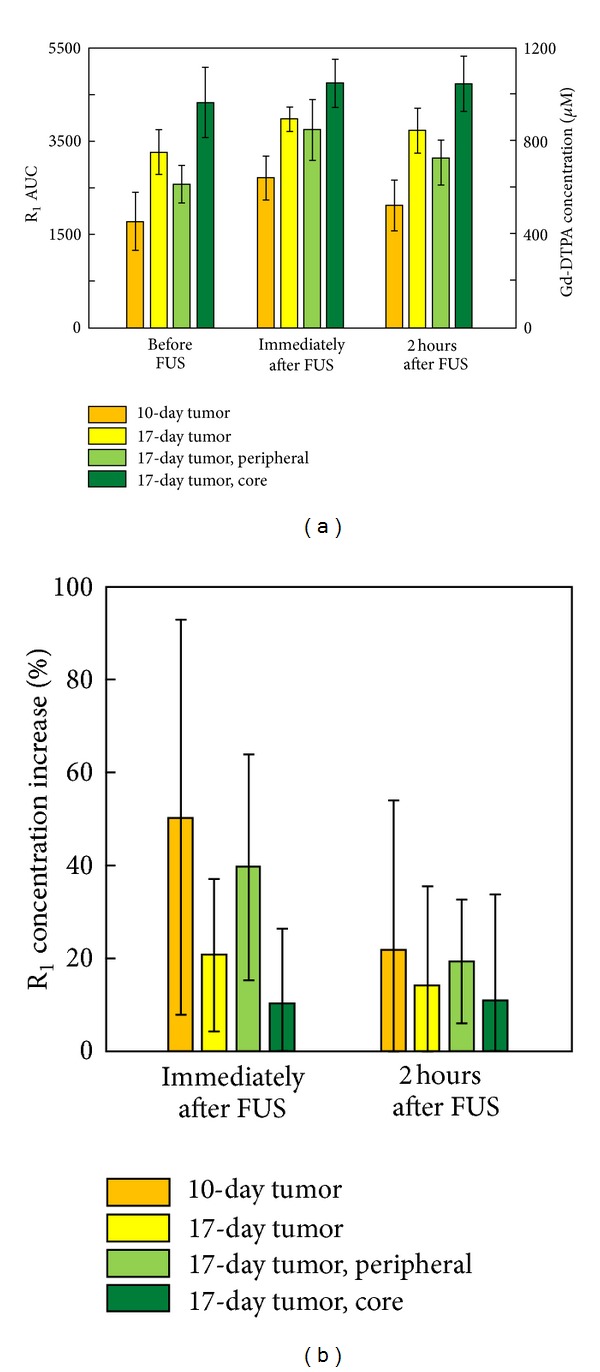
(a) R_1_ AUC analysis of the core and peripheral subregions of 17-day tumor. (b) R_1_ AUC increase relative to time slot I.

Previous reports showed that the tumor core consists of a bulky necrotic mass without functional vasculature, whereas the tumor periphery maintains a high degree of vasculature structure [28, 29]. We therefore hypothesized that microbubble-enhanced FUS exposure would have a bigger effect on enhancing permeability in the peripheral tumor. We further divided the 17-day tumor animals into core and peripheral subregions and then repeated the MRI AUC analysis (Figure 10). We observed that, after FUS exposure, the 17-day peripheral tumor showed a similar trend to the 10-day tumor, which showed a nearly 1.7-fold of instantaneous increase and 50% accumulation increase in Gd-DTPA, and the permeability dropped significantly two hours after FUS exposure to about half (20%), which is similar to 10-day tumor. In contrast, the 17-day tumor core mimicked the behavior of the undivided 17-day tumor, accumulating high levels of Gd-DTPA both before and after FUS exposure (AUC increase of only 10% after FUS exposure) (Figure 10). This relatively low increase in the AUC persisted when the tumor core was re-evaluated at 2 hours after FUS exposure (9%). These observations suggest that the FUS-BBB opening can provide the most pronounced drug delivery enhancing effect on tumor peripheral or tumors with high-vascularity stage tumors, yet only provides limited effects on bulky and necrotic tumors.

## 4. Discussion

This study demonstrated the pharmacodynamic characteristics of small-molecule leakage at various stages of tumors after application of microbubble-enhanced FUS to open the BBB. We analyzed two small molecules with similar molecular weights to obtain complimentary data on pharmacodynamic behavior. Gd-DTPA was used to provide contrast in MRI and for semiquantitative verification of biodistribution *in vivo*, and EB dye was used as a measure of drug accumulation after animal sacrifice. These two molecules, which normally do not enter the brain parenchyma from the bloodstream, could potentially be used as surrogate markers for drug delivery. Although the dynamic distribution of Gd-DTPA may differ from that of Evans blue, we demonstrated that the AUC accumulation of Gd-DTPA analyzed by MRI was highly correlated with EB accumulation in the brain (*r* ^2^ = 0.8897), implying that MRI AUC analysis of Gd-DTPA could predict the concentration of EB accumulating in the brain, and may thus have the potential to predict the pharmacodynamic behavior and biodistribution of other therapeutic agents. 

This study employed high-temporal-resolution dynamic CE-MRI that could be utilized for small-molecule *in vivo* distribution and semiquantification, as attempted in previous studies [30, 31]. The unique features provided by dynamic CE-MRI include the capability of rapid evaluation and high spatial resolution, as well as kinetic analysis to evaluate tumor perfusion [30]. Positron emission tomography (PET) has also been used for pharmacological studies in several tumor types [32, 33]. However, potential limitations of PET may include its limited imaging resolution and complexity of radiotracer synthesis. In small tumors, partial volume effects may be significant if the tumor size is less than twice the resolution of the scanner [34]. MRI methods provide the advantage of having good spatial resolution equal to that of corresponding morphologic images. In addition, MRI is minimally invasive and poses little risk to patients. We used voxels of about 0.26 × 0.26 × 1 mm^3^ to construct images sufficient for small-animal analysis. On the other hand, PET relies on radiolabeled molecules that bind to receptors to allow absolute quantification by detection of isotopes. PET may also be limited by its high cost, limited availability of radiotracer, and the need for a cyclotron as well as onsite radiochemistry for radioisotope production [35]. PET involves comprehensive conjugation of radiotracers and specific tailor-made molecules limiting its general use for pharmacodynamic analysis. Although Gd-DTPA cannot be directly conjugated to therapeutic molecules, the detection of coadministered Gd-DTPA by CE-MRI is highly correlated with targeted molecules, providing an excellent tool for monitoring vasculature and evaluating tissue/tumor permeability at high temporal/spatial resolution, suggesting its continued usefulness for pharmacodynamic analysis of brain drug delivery.

Tumor tissues are known to have high permeability due to the presence of large endothelial cell gaps, incomplete basement membrane, and the relative lack of pericyte or smooth muscle association with endothelial cells [36, 37]. In addition, the network of vasculature in solid tumors is markedly different from the normal hierarchical branching patterns and contains leaky vessel structures. Variations in permeability are also associated with the tumor grade as well as various neoplastic effects that could disrupt the BBB [38]. In this study, we observed that tumors with different levels of progression showed different characteristics of blood-vessel permeability and small-molecule accumulation. We found that the AUC_80 min⁡_ in small tumors was 452 ± 122.5 *μ*M, whereas in large tumors it reached 754 ± 48.3 *μ*M. Moreover, we confirmed that FUS-BBB opening provided a 50% enhancement of accumulation of Gd-DTPA in small brain tumors and a 40% enhancement at the large tumor periphery, implying that FUS-BBB opening is an effective approach to increase brain-tumor permeability and therefore enhance delivery of therapeutic molecules.

 Histological examination by HE staining showed that smaller (10-day) tumors had well-ordered vasculature with fewer abnormal endothelial cell gaps (Figures 9(a) and 9(b)). The blood vessel density in small tumor was lower, resulting in less Gd-DTPA and EB accumulation. In contrast, 17-day tumor tissues contained more large fenestrae (Figures 9(e) and 9(f)), consistent with previous pathological findings that high-grade brain tumors contain neovasculature and apoptotic tumor cells, leading to hyperpermeability [39]. These pathological changes are consistent with the increased Gd-DTPA and EB accumulation that we observed in 17-day tumor tissues before sonication.

Although AUC_80 min⁡_ correlated well with the pharmacodynamic behavior of another small molecule (EB), Gd-DTPA accumulation can be very different even under the same FUS exposure conditions, for example, varying from 365.2 to 900 *μ*M (Figure 7(b)). These large variations were likely due to differences in skull thickness and angle of incidence between the FUS beam and the skull surface among the animals [40], and the presence of standing waves produced in the skull cavity that alter the peak pressure at the target position and thus the level of BBB opening [41]. Since FUS-BBB opening may vary substantially, it is essential to perform an AUC analysis during CE-MRI to monitor small-molecule delivery into the brain for individual subjects and targets. 

## 5. Conclusion

 In this study, we characterized the dynamics of BBB opening in normal and tumor tissues using DCE-MRI with Gd-DTPA contrast agent, and related them to the concentrations of Evans Blue determined from tissues after sacrifice. The concentrations of the surrogate tracer (Gd-DTPA) and EB dye showed a strong linear correlation. With this dynamic information of tumor permeability, the pharmacodynamic model can be modified to eventually take into account parameters that affect drug delivery over time. Tumor peripheral or high-vascular tumor may have the most significant benefit on blood-brain or blood-tumor permeability increase, which gives critical information when intending to apply FUS for brain drug enhanced delivery. We hope to use such pharmacodynamic predictions along with FUS-induced BBB opening to develop a method for image-guided drug delivery that can estimate the amount of drug that will be delivered to tissues at each time point.
